# Companion Animal Type and Level of Engagement Matter: A Mixed-Methods Study Examining Links between Companion Animal Guardianship, Loneliness and Well-Being during the COVID-19 Pandemic

**DOI:** 10.3390/ani11082349

**Published:** 2021-08-09

**Authors:** Heather Clements, Stephanie Valentin, Nicholas Jenkins, Jean Rankin, Nancy R. Gee, Donna Snellgrove, Katherine A. Sloman

**Affiliations:** 1 School of Health & Life Sciences, University of the West of Scotland, Paisley PA1 2BE, UK; j.rankin@uws.ac.uk; 2 School of Health & Life Sciences, University of the West of Scotland, Glasgow G72 0LH, UK; stephanie.valentin@uws.ac.uk; 3 School of Education & Social Sciences, University of the West of Scotland, Glasgow G72 0LH, UK; nick.jenkins@uws.ac.uk; 4 Center for Human–Animal Interaction, School of Medicine, Virginia Commonwealth University, Richmond, VA 23298, USA; Nancy.Gee@vcuhealth.org; 5 WALTHAM Petcare Science Institute, Waltham-on-the-Wolds LE14 4RT, UK; Donna.Snellgrove@effem.com

**Keywords:** human–animal interaction, ornamental fishes, COVID-19, mixed methods, loneliness, mental well-being

## Abstract

**Simple Summary:**

Companion animals (pets) may reduce loneliness and promote the well-being of their guardians (owners). This is important in the context of the COVID-19 pandemic, as companion animal guardians may be less negatively affected by the pandemic. This research examined the influence of companion animals, and specifically ornamental fishes, on mental well-being and loneliness during the pandemic. Data were collected via an online survey and interviews with companion animal guardians. Companion animal guardianship alone was not linked to loneliness or well-being during the pandemic, but there was evidence that people who interacted more with their dogs (and to a lesser extent cats) were lonelier and had poorer well-being; possibly, these individuals spent more time with their dogs/cats because they were more isolated. Open-ended survey responses and interview data identified that most people felt their companion animals were a positive influence during the pandemic, but ornamental fishes were perceived as having a less positive effect than other companion animals, possibly because they cannot provide comfort via physical touch. Consistent with past research, these findings indicate that people believe their companion animals positively influenced their lives during the pandemic, but there is a lack of quantitative evidence to support these beliefs.

**Abstract:**

To reduce the spread of COVID-19, countries worldwide placed limitations on social interaction, which is anticipated to have severe psychological consequences. Although findings are inconsistent, prior research has suggested that companion animals may positively influence human well-being and reduce loneliness. In the context of COVID-19, this has important implications, as companion animal guardians may be less negatively affected by the pandemic. The primary aim of this research was to investigate the influence of companion animals on mental well-being and loneliness during the pandemic, with specific interest in the role of ornamental fishes. A mixed-methods study was conducted, using an international sample. Quantitative data were collected via an online survey (*n* = 1199) and analysed using robust hierarchical multiple regression analyses; the influence of level of engagement with companion animals was examined for dogs, cats and ornamental fishes. There was no evidence that companion animal guardianship was associated with loneliness and mental well-being during the pandemic but spending more time engaging physically or socially with dogs (and to a lesser extent cats) was generally associated with poorer outcomes. Qualitative data were collected through open-ended survey responses (*n* = 757) and semi-structured interviews (*n* = 25) and analysed using reflexive thematic analysis. Two themes were developed—one related to companion animals as providers of social and emotional support, and the other to companion animals as providers of purpose and perspective. Concerns regarding the impact of the pandemic on animal welfare were also identified. Compared to other animal types, more participants expressed indifference regarding the impact of their fishes on their well-being during the pandemic, possibly because fishes cannot provide comfort via physical touch. The findings of this study reflect the wider field of human–animal interaction; although qualitative data suggest guardians believe their companion animals are a positive influence in their lives, there is little convincing quantitative data to support these beliefs. This highlights the need to refine theories regarding which aspects of companion animal guardianship may influence human well-being; the findings from this research may be useful in the refinement of such theories.

## 1. Introduction

On 11 March 2020, the World Health Organisation (WHO) declared the COVID-19 outbreak to be a pandemic. The impact of this pandemic is catastrophic; at the time of writing, over 187 million cases and 4 million deaths have been recorded globally [1]. To inhibit the spread of the virus, countries worldwide adopted strategies to limit physical contact between individuals from different households; this included nationwide lockdowns, travel restrictions and social distancing [2].

The psychological consequences of the pandemic are potentially severe [3]. It is well established that social isolation and loneliness are detrimental to physical and mental health, with a risk of premature mortality comparable to that of factors such as obesity or substance abuse [4,5]. Worries related to the pandemic are also a cause of psychological distress. For example, one study conducted during the first six weeks of lockdown in the United Kingdom (UK) identified two aspects of anxiety associated with COVID-19—disease anxiety related to catching or transmitting the virus, and consequence anxiety related to the longer-term impacts of the pandemic, such as in relation to the economic cost [6]. Numerous studies from various countries have already reported psychological issues associated with the early stages of the pandemic [7,8,9,10].

Companion animals are widely believed to promote positive well-being. For instance, companionship and emotional support were among the most common reasons given for keeping companion animals among a sample of dog and cat guardians [11], while qualitative findings have indicated that companion animals are considered important sources of mental health support [12,13,14,15]. On the other hand, several cross-sectional studies have found no association, or a negative association, between companion animal guardianship and mental or physical health [16,17,18,19,20,21]. There is also no convincing evidence that companion animals directly influence loneliness [22], although an indirect effect may occur via social facilitation [23,24]. Furthermore, human–animal interaction (HAI) research frequently suffers from methodological issues, such as inadequate sample sizes, high levels of heterogeneity, and a lack of blinding [25,26,27,28], plus participants are likely to have positive attitudes towards companion animals, and so findings may not be generalisable [27]. Thus, the influence of companion animals on loneliness and mental well-being remains unclear, but if an effect exists, this may have important implications regarding the psychological impact of the COVID-19 pandemic; companion animal guardians may be less negatively affected in comparison to non-guardians.

However, companion animals may also have detrimentally impacted well-being during the pandemic. Previous research has highlighted negative aspects of keeping companion animals, such as the financial cost and the burden associated with their care [13]. It is possible that these negative aspects were amplified during the pandemic, for instance if restrictions on movement or loss of income impacted the ability of guardians to care for their companion animals. There is also evidence that some guardians are concerned about the potential of companion animals to transmit or contract the SARS-CoV-2 virus [29]. Therefore, the primary aim of this research was to investigate the relationship between companion animal guardianship, and mental well-being and loneliness during the COVID-19 pandemic; at conception of this study, no published research on this topic was identified.

A secondary aim of this research relates to a broader question in the field of HAI; how any effects associated with companion animal guardianship are influenced by the type of companion animal. Much research has focused on animals that interact with humans in a physical or social manner, namely dogs, cats and horses. Comparatively few studies have examined the impact of other animal types, such as ornamental fishes [30]. Fishes represent a significant proportion of companion animals, for example they are the third most popular companion animal in the UK and are by far the most numerous, as one home aquarium may contain many fishes (www.pfma.org.uk, accessed on 26 February 2021). Only one study to date has examined the impact of fish guardianship on loneliness, and no effect was observed [31]. However, qualitative evidence has demonstrated that some fish guardians believe their fishes provide companionship, along with other psychological benefits such as relaxation [32]. Fishes have also been associated with positive outcomes such as reduced agitation, increased food intake and improved body mass among people with dementia [33,34,35], reduced anxiety among students preparing for an impromptu presentation [36], and improved mood among visitors to a public aquarium [37]. Thus, the secondary aim of this study was to investigate the specific relationship between keeping ornamental fishes and mental well-being and loneliness during the pandemic.

As the comparison of companion animal guardians and non-guardians may represent an oversimplification of the relationship between companion animal guardianship and well-being [38,39], factors such as sociodemographic characteristics are now widely accounted for in cross-sectional HAI research. Less well understood is how the type and amount of engagement between an individual and their companion animal may influence the relationship between companion animal guardianship and well-being [26,38]. Thus, the current study examined how these relationships may be influenced by the amount of time participants spent engaging in specific behaviours with their companion animal.

Finally, as it is possible that neither quantitative nor qualitative methods are sufficient to understand the relationship between companion animal guardianship and well-being when used in isolation, the current study used an embedded mixed-methods approach. The purpose of using this approach was to obtain qualitative data that could support the interpretation of quantitative findings.

## 2. Materials and Methods

### 2.1. Ethical Approval

This study was approved by the School of Health and Life Sciences ethics committee at the University of the West of Scotland (Ref:12045), and the Mars Research Review Board.

### 2.2. Design and Procedure

**Figure 1:**
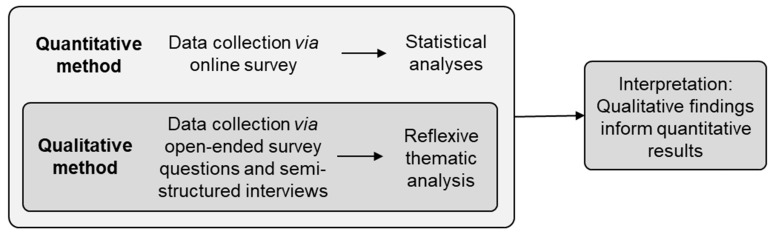
Embedded mixed-methods design.

This study used an embedded mixed-methods design [40], with the qualitative component embedded within the larger quantitative study (Figure 1). A convergent approach was used. Quantitative data were collected from June to November 2020 via an online, cross-sectional survey, and qualitative data were collected from June 2020 to January 2021 via open-ended survey questions and in-depth interviews; all participants who completed both components completed the survey prior to the interview. Both datasets were analysed independently, with the qualitative findings used to inform the interpretation of the quantitative results.

### 2.3. Participants and Recruitment

Survey participants (*n* = 1199) were an international sample of adults (18+ years) proficient in the English language. The sample comprised mainly individuals from the UK (51%) and USA (32%). Participants were recruited through opportunity sampling (e.g., via social media), with fish-specific organisations/networks purposively targeted. Interview participants (*n* = 25) were recruited either by them contacting the researcher following completion of the survey, or via the same process described above; they comprised individuals from either the UK (84%) or the USA (16%). Both companion animal guardians and non-guardians were eligible to participate in the survey, whereas only companion animal guardians could take part in an interview. Very few participants kept ornamental fishes as their only companion animal, thus data from fish guardians represent the experiences of those who keep ornamental fishes in addition to other companion animals. Informed consent was obtained from all participants prior to their participation.

### 2.4. Materials

#### 2.4.1. Survey

The survey consisted of a mix of open- and closed-ended questions split over six sections, as described in Table 1.

#### 2.4.2. Interviews

A semi-structured interview schedule was used. Demographic information was collected via closed questions, after which participants were asked to tell the researcher “a little bit” about their companion animals. The rest of the interview focused on their experiences of being a companion animal guardian during the pandemic. All interviews were conducted remotely via phone (*n* = 7) or video conferencing (*n* = 18), and were audio recorded with the permission of the participant.

### 2.5. Data Analysis

#### 2.5.1. Quantitative Data Management

Survey data were checked for accuracy and completeness prior to analysis; three participants provided inconsistent answers and so their data were removed. Ambiguous responses and those of “prefer not to say” were recorded as missing data but did not exceed 2% of responses per item. Where data were missing for the standardised assessments (<2% per scale), total scores were not calculated. Several demographic variables had low response rates for one or more categories. Where there was reasonable justification, these categories were combined prior to analysis (e.g., for country of residence “Australia or New Zealand” (*n* = 39) and “Canada” (*n* = 19) were collapsed into the category of “other” country). Where there was no reasonable justification, the original categories were retained unless cell sizes were too small to be included in the analyses (e.g., the category of “partner, not living together” (*n* = 72) for marital status was retained, but genders other than male or female (*n* = 6) could not be included).

The response categories for the social and lifestyle variables were also condensed to reduce the number of parameters in the analyses and provide greater balance in group sizes. For each variable, the median was identified and responses within the corresponding category were assigned a value of ‘average’. Responses for the categories above and below the median were recorded as ‘more than average’ and ‘less than average’, respectively. For example, for frequency of exercise outside the home the median fell within the category “A few times a week” so these responses were recorded as average; responses of “Never” to “Once a week” were recorded as less than average, and responses of “Once a day” or “More than once a day” as more than average. Where the median fell within the highest or lowest category, that variable was not included in the analyses. Variables relating to the level of engagement with companion animals (e.g., time spent walking dogs) were condensed using the same procedure. Due to wide variation in employment status, participants were categorised as either “working” or “not working” during the pandemic.

#### 2.5.2. Companion Animal Guardianship, Mental Well-Being and Loneliness

Separate hierarchical multiple regression analyses (as in [46]) were conducted to determine whether companion animal guardianship was related to scores on the standardised assessments of depression, anxiety, stress, mental well-being and loneliness. The model had three stages. Demographic characteristics were entered in stage one; it was not possible to include ethnicity as the sample was predominantly white (*n* = 1096, 91%). Social and lifestyle factors were entered at stage two; bivariate correlations were used to determine which variables significantly correlated with at least one dependent variable, and these variables were included in the model. Companion animal guardianship was entered in the final stage as separate dichotomous predictors for whether participants kept any type of companion animal, and whether they kept dogs, cats, fish or other types of companion animal (yes/no for each).

Data were analysed using R version 4.0.0 [47]. There was no evidence of multicollinearity, but in all cases, diagnostics indicated that assumptions were violated due to the presence of influential data points and non-normality of residuals. As robust analyses are recommended for data which violate model assumptions [48], robust regression analyses were conducted via the *“lmrob”* function of the *“robustbase”* package [49], using the default settings recommended by Koller and Stahel [50]. At each stage a Wald-type test statistic was used to assess whether the inclusion of the additional predictors resulted in a significant change in robust deviance compared to the reduced model; *p*-values < 0.05 were deemed to be statistically significant.

Multiple scales were used to measure loneliness, but no differences were found with regard to influence of companion animal guardianship across these measures, so only results pertaining to the UCLA-LS are reported.

#### 2.5.3. Level of Engagement with Companion Animals, Mental Well-Being and Loneliness

To examine the influence of level of engagement between a guardian and their companion animal, further robust multiple regression analyses were conducted for the subsets of participants who kept dogs, cats and fishes. Each model included both general and species-specific engagement variables, and whether the participant was the primary caregiver for their companion animal (yes, no or responsibility equally shared with another). All predictors were entered into the models simultaneously, otherwise the analyses were conducted as described above.

#### 2.5.4. Qualitative Data Analysis

Data analysis was conducted using NVivo 12 Pro [51]. Interview data were combined with responses to the survey question “In what ways, if any, do you feel your companion animals have influenced your mental well-being during the pandemic?” (*n* = 757), and analysed using reflexive thematic analysis [52,53,54]. An essentialist approach was followed, assuming that participants’ accounts reflected reality, and seeking to understand these experiences to deepen understanding of the quantitative findings. The analysis was conducted by HC; KS read the entire dataset and the final report to ensure the analysis provided an accurate and complete representation of the data.

Familiarisation was achieved through verbatim transcription of the interviews and active re-reading of the dataset. Generation of codes was based on the semantic content of the data and followed a predominantly inductive approach; potential codes identified during familiarisation formed the basis of the analysis, but the researcher coded openly for any relevant features of the dataset. Following initial coding, themes were constructed by organising codes into groups to identify “patterns of shared meaning across the dataset” [53] (p. 592); thematic maps were used to support this process and visualise the structure of the analysis. Candidate themes were then ‘tested out’ against the data; all extracts coded onto each theme were examined to determine whether they formed a coherent pattern, and brief textual descriptions were produced and compared to identify areas of overlap. Adjustments were made as needed. Finally, the entire dataset was reviewed to ensure the themes accurately and fully represented the data. Each of the final themes represents a patterned response within the data that captures something of importance to the research question; they were not selected based on prevalence alone.

Data were analysed across all types of companion animal collectively. However, in-line with the objective to focus on ornamental fishes, extracts were chosen to represent companion animal guardianship in general, with additional quotations from fish guardians provided where relevant.

## 3. Results

### 3.1. Sample Characteristics

#### 3.1.1. Survey

Survey data were available for 1199 participants; 1159 (97%) responded to the question “Do you have any companion animals (pets)?” and 1069 (89%) reached the end of the survey. Most participants (*n* = 1005, 84%) kept at least one companion animal, with 42% (*n* = 419) of these individuals keeping a mix of companion animal types. Dogs were the most popular species both overall (*n* = 661, 66%) and where they were the only type of companion animal kept in the household (*n* = 329, 33%); cats were the second most popular (total *n* = 469, 47%; single species *n* = 200, 20%). Fewer individuals kept fishes (*n* = 218, 22%), small mammals (*n* = 87, 9%), exotic animals (*n* = 77, 8%), birds (*n* = 65, 6%) or other types of companion animal (*n* = 47, 5%), and in all cases 2% or less were kept as the only species. “Other” companion animals included horses, ponies or farm animals, insects/invertebrates, and wild birds. With regard to ornamental fishes, most participants kept their fishes in tanks (*n* = 171, 78%), or tanks and ponds (*n* = 33, 15%); only 10 participants (5%) kept fishes in ponds alone (four participants did not state whether their fishes were kept in tanks or ponds). Survey sample characteristics for companion animal guardians and non-guardians are summarised in Table 2; a breakdown of sample characteristics by type of companion animal can be found in Table S1.

#### 3.1.2. Interviews

Twenty-five participants took part in an interview. Most identified as female (*n* = 22, 88%); 10 (40%) were aged 18–34 years, with slightly fewer aged 35–50 years (*n* = 7, 28%) or 51–69 years (*n* = 8, 32%). All resided in the UK (*n* = 21, 84%) or the USA (*n* = 4, 16%) and most lived with other people (*n* = 21, 84%). Dogs and cats were the most popular companion animals (*n* = 13 for both, 52%). Seven participants (28%) kept fishes, four (16%) kept small mammals, and three (12%) kept exotic animals; no participants kept birds or other companion animals. Some individuals did report keeping invertebrates, but these were always kept alongside fishes. Eleven participants (44%) kept a mix of companion animal types; all participants who kept fishes kept at least one other type of companion animal. Fourteen participants (56%) were the primary caregiver for at least one companion animal, and all other participants shared this responsibility with another person.

### 3.2. Quantitative Findings

**Figure 2:**
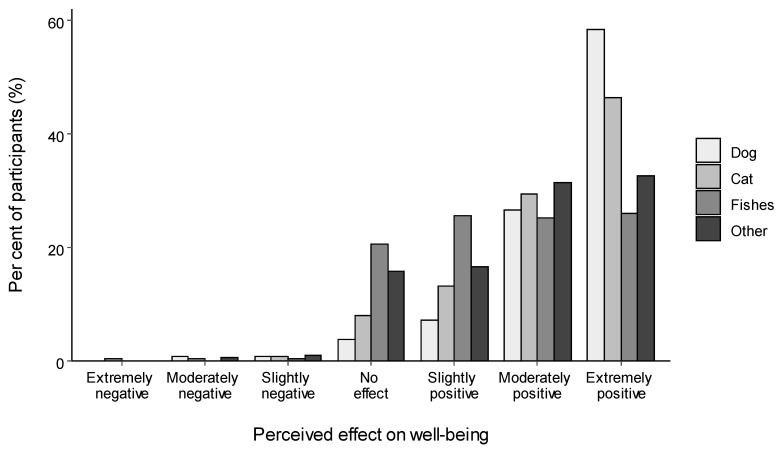
The percentage of participants who responded with each category across the scale by type of companion animal. ‘Other companion animal’ is inclusive of small mammals, exotic animals, birds and otherwise unspecified companion animals (e.g., horses). Dogs *n* = 661, cats *n* = 469, fish *n* = 218, and other companion animal *n* = 276.

Participants who kept companion animals overwhelmingly rated them to have had a positive effect on their well-being during the pandemic (Figure 2). Most participants believed their dogs and cats had an extremely or moderately positive effect on their well-being, while fewer individuals believed they had a slightly positive effect or no effect at all. To a lesser extent, the same patten of results was observed for guardians of other companion animals, whereas for fishes there was a more even split across these four categories. For all animal types, only a very small minority of participants rated their companion animals as having had any degree of negative effect on their well-being (<2%).

Eighteen per cent of participants had experienced issues obtaining pet care supplies and 14% reported problems accessing veterinary treatment; 13% reported additional negative impacts associated with the pandemic (e.g., reduced frequency of dog walking, no access to professional grooming services). Twelve per cent of participants (*n* = 125) reported adopting or purchasing a new companion animal during the pandemic, with around a quarter (24%) agreeing that the pandemic was instrumental in their decision to adopt. Most participants (92%) reported being not at all concerned about contracting SARS-CoV-2 from their companion animals; 7% were a little concerned and less than 1% were somewhat or extremely concerned. More individuals reported being a little (20%), somewhat (6%) or extremely (1%) concerned about passing SARS-CoV-2 onto their companion animals. However, most (73%) were not at all concerned about this possibility.

#### 3.2.1. Companion Animal Guardianship, Mental Well-Being and Loneliness

Correlations between participants’ scores on the standardised assessments and their subjective ratings of how the pandemic had influenced their loneliness and mental well-being ranged from medium to large in size (*r*_s_ = 0.36–0.51, *p* < 0.001 in all cases). This suggests that scores on the standardised assessments likely reflected a combination of pre-existing mental states and pandemic-related changes.

Full results of the robust hierarchical regression analyses are shown in Table S2. Participants’ demographic characteristics explained between 7.1% and 12.9% of variance in their scores on the standardised assessments. In all cases, this was a statistically significant improvement in the model fit (χ^2^ (12) = 74.94–150.85, *p* < 0.001 in all cases). Social and lifestyle factors explained an additional 2.6% to 3.6% of variance; again, the model fit was significantly improved for all dependent variables (χ^2^ (8) = 27.66–42.54, *p* < 0.01 in all cases). The addition of companion animal guardianship factors did not significantly improve the model fit for any dependent variable (χ^2^ (5) = 1.40–9.88, *p* > 0.05 in all cases). Dog guardianship was found to be significantly associated with depression, and guardianship of other companion animals was found to be significantly associated with anxiety. However, in both cases there was no overall change in robust deviance, suggesting that these associations emerged because dog and other companion animal guardianship shared variance with a predictor or predictors already included in the model.

#### 3.2.2. Level of Engagement with Companion Animals, Mental Well-Being and Loneliness

The results of the robust regression analyses are given in Table S3. Level of engagement with dogs explained between 4.8% and 7.1% of variance in scores on the standardised assessments, and in all cases this was a statistically significant improvement in the model fit (χ^2^ (12) = 28.19–48.03, *p* < 0.01 in all cases). With respect to the contribution of individual predictors, spending less time than average talking to dogs was associated with higher mental well-being ($\hat{\beta }$ = 2.41, SE = 1.22, *t* = 1.97, *p* < 0.05). Lower mental well-being ($\hat{\beta }$ = −2.22, SE = 1.08, *t* = −2.07, *p* = 0.039) and higher depression ($\hat{\beta }$ = 1.78, SE = 0.88, *t* = 2.03, *p* = 0.043) were associated with a greater than average amount of time spent petting dogs, while spending less time than average petting dogs was associated with lower anxiety ($\hat{\beta }$ = −1.59, SE = 0.58, *t* = −2.74, *p* = 0.006). Walking dogs for less time than average per day was associated with both higher anxiety ($\hat{\beta }$ = 1.38, SE = 0.46, *t* = 3.02, *p* = 0.003) and higher loneliness ($\hat{\beta }$ = 0.41, SE = 0.17, *t* = 2.35, *p* = 0.019); loneliness was also associated with less time than average spent undertaking ‘other’ activities with one’s dog ($\hat{\beta }$ = 0.49, SE = 0.20, *t* = 2.40, *p* = 0.017). Finally, being the primary caregiver for a dog, or sharing this responsibility with another person, was associated with more positive scores across all dependent variables relative to not being the primary caregiver (*p* < 0.05 in all cases).

The model for level of engagement with cats was a statistically significant improvement on the null model for depression, anxiety, and stress (χ^2^ (10) = 20.48–23.39, *p* < 0.05 in all cases). These models explained between 4.5% and 5.2% of variance in the relevant dependent variable. Spending more time than average talking to cats was associated with lower anxiety ($\hat{\beta }$ = −1.76, SE = 0.75, *t* = −2.35, *p* = 0.019) whereas spending less time than average was associated with lower depression ($\hat{\beta }$ = −4.29, SE = 1.15, *t* = −3.73, *p* = <0.001); lower stress was associated with spending both more ($\hat{\beta }$ = −3.01, SE = 1.35, *t* = −2.23, *p* = 0.026) and less ($\hat{\beta }$ = −3.65, SE = 1.25, *t* = −2.92, *p* = 0.004) time than average talking to cats. For mental well-being and loneliness, level of engagement with cats did not significantly improve the model fit (mental well-being, χ^2^ (10) = 14.24, *p* = 0.162; UCLA-LS, χ^2^ (10) = 14.83, *p* = 0.139).

The model for level of engagement with fishes did not result in a significant improvement in model fit for any of the dependent variables (mental well-being, χ^2^ (10) = 5.37, *p* = 0.865; loneliness, χ^2^ (10) = 9.45, *p* = 0.450; depression, χ^2^ (10) = 5.33, *p* = 0.868; anxiety, χ^2^ (10) = 7.34, *p* = 0.693; stress, χ^2^ (10) = 9.46, *p* = 0.489).

### 3.3. Qualitative Findings

Two themes were developed from the data. “Companion animals as providers of social support” related to the notion that companion animals offered an alternative to interpersonal connections during a time of isolation. “Companion animals as providers of purpose and perspective” related more to the practicalities of being a companion animal guardian, and how this helped participants retain a sense of normality and feel useful when they may have otherwise become dispirited. There was also evidence that animal welfare may have been influenced by the pandemic, but these data did not relate to the current research question and so are not reported here. Example quotations are provided in Table 3 and Table 4.

#### 3.3.1. Theme 1: Companion Animals as Providers of Social Support

“It meant never being alone”

Most participants felt their companion animals had provided comfort and support, both prior to and during the pandemic. This support was experienced at two levels. At a basic level, participants were comforted by the mere presence of a companion animal as another living being in their home. At a deeper level, companion animals provided psychological and emotional support, helping their guardians cope with the uncertainty caused by the pandemic. Individuals experiencing a greater degree of isolation particularly appreciated the support provided by their companion animals, such as those who lived alone or had switched to remote working during the pandemic.

Despite being isolated from friends, family or colleagues, having companion animals meant that participants were never truly alone; they were surrogates for interpersonal contact when connections with other humans were restricted. Although the quantitative results showed no evidence of a relationship between companion animal guardianship and loneliness, some participants clarified that their companion animals could not fully replace the need for human-to-human connections. Therefore, the comfort and support provided by companion animals may have been insufficient to alleviate feelings of loneliness to an extent that it was observable via quantitative assessments.

“A substitute for human talk and touch”

Given the need to maintain physical distance from other humans, it is unsurprising that cuddling and other forms of physical affection were an important aspect of the support provided by companion animals during the COVID-19 pandemic. Many participants also reported talking to their companion animals, and their non-verbal responses provided comfort by showing that somebody was listening. Again, these effects were of particular importance to individuals who were more isolated from their social networks, suggesting that where human-to-human interactions were impossible due to pandemic-related restrictions, companion animals provided an accessible alternative. This corresponds with the quantitative finding that a higher level of engagement with dogs, and to a lesser extent cats, was associated with poorer outcomes for some dependent variables; individuals experiencing greater isolation may have simultaneously experienced poorer mental states and been more inclined to seek support from their companion animals. Furthermore, if the comfort and support provided by companion animals was dependent on these physical and social interactions, it would explain why participants were less positive about the impact of ornamental fishes on their well-being during the pandemic; as put succinctly by one participant, it is “hard to hug a fish” (Survey, Participant 171).

“Bridging interpersonal connections during social distancing”

Companion animals also benefitted their guardians by facilitating interpersonal connections. Even during the tightest restrictions, leaving the house was permitted for reasons related to animal welfare, such as to walk dogs or attend to horses. This often led to brief socially-distanced interactions that were vastly appreciated during the period of isolation. Correspondingly, the quantitative results indicated that walking dogs for less time than average each day was associated with higher loneliness; possibly, those who spent less time walking their dogs had fewer opportunities for these brief social encounters.

Within existing interpersonal relationships, activities such as playing with or exercising companion animals were an opportunity to connect, particularly when other social or leisure activities were unavailable. Even when communication took place via electronic methods, companion animals acted as social catalysts by providing a topic of conversation. However, these benefits may not have been limited to companion animal guardians. Some participants noted that friends, family and strangers were interested in hearing about, or interacting with their companion animals, and it is unclear whether these individuals kept companion animals themselves. As the quantitative results did not account for human–animal interaction outside of the companion animal guardianship dynamic, these interactions could have confounded the results, particularly as participants of a survey about companion animals are likely to have positive attitudes towards animals, irrespective of their current guardianship status.

#### 3.3.2. Theme 2: Companion Animals as Providers of Purpose and Perspective

“They help maintain a normal routine”

Being unable to understand the concept of a pandemic, companion animals continued to adhere to their usual schedules, and expected activities such as meals and walks to occur at specific times of day. As participants recognised their responsibility to their companion animals, they also continued to adhere to these routines. This was beneficial as it added structure to their day and allowed them to experience something which resembled normal life; ornamental fishes were particularly beneficial in this regard, as regular tank maintenance was essential for fish welfare (see Table 4 for supporting quotations). The timing of data collection may explain why these benefits are not reflected in the quantitative findings. Data collection began around three months after COVID-19 was declared a pandemic by the WHO. At this time, many individuals had recommenced work outside the home, while those working remotely will have had the opportunity to develop new working routines. Had data collection commenced earlier during the pandemic, these effects may have been evident as participants had not yet adjusted to the “new normal”.

Exercise routines were also appreciated, and many participants felt that without the need to walk their dogs they would have lacked the motivation to engage in regular physical activity. These routines also gave participants an opportunity to be outdoors, which was greatly valued during the restrictions imposed due to the pandemic. There was quantitative evidence that having primary or shared responsibility for a dog was associated with more positive scores for all dependent variables. As having a greater level of responsibility for a dog presumably increases the likelihood of walking that dog, this supports the idea that the maintenance of exercise routines was an important benefit of dog guardianship during the pandemic.

“Something good to focus on”

Many participants indicated that their companion animals provided a source of positive distraction throughout the pandemic. Companion animals not only helped to take their guardians’ minds off negative thoughts associated with the pandemic, but also provided a much-needed source of pleasure; their antics gave their guardians a reason to smile. Among participants who kept ornamental fishes, watching home aquaria was frequently cited as a beneficial activity; the movement and behaviour of the fishes in the tank captured participants’ attention, distracting them and inducing relaxation. These findings suggest that the effect of companion animals on well-being during the pandemic may have been relatively transient; a guardian may have experienced relief from negative mental states while actively watching or interacting with their companion animal, but it is unclear how long this effect was retained once the interaction concluded. As the assessments used in the current study related to well-being over the previous two weeks, they unfortunately provide no insight into the role of companion animals as providers of momentary relief.

“A reason to keep going”

The responsibility of being a companion animal guardian gave many participants a sense of purpose. For those experiencing employment issues, caring for a companion animal was a source of the meaningful activity, whereas others were motivated to ‘carry on’ by the knowledge that their companion animal was entirely dependent on them. Thus, companion animal guardianship may not have protected against the negative psychological impact of the pandemic, but may play a role in how participants cope with these negative consequences going forward.

Conversely, many participants expressed concern about whether the imposed restrictions would impact their ability to care for their companion animals, particularly in relation to obtaining supplies and veterinary treatment. Although for most these concerns were not realised (a finding corroborated by the quantitative data), several participants expressed that this was an added stress during an already stressful time. Some participants also expressed greater concern regarding the impact of the pandemic on their companion animals, than on their own well-being. This suggests any benefits associated with being a companion animal guardian during the pandemic may have been negated by additional stresses associated with this responsibility. Thus, the quantitative analyses may have failed to find evidence of any associations between companion animal guardianship and loneliness and mental well-being during the pandemic because the relationship between these variables is more complex than was accounted for in the statistical models.

## 4. Discussion

This study aimed to examine the relationship between companion animal guardianship and loneliness and mental well-being during the COVID-19 pandemic. Quantitative data analyses found no evidence of a linear relationship between keeping companion animals and loneliness or mental well-being, either for all animal types collectively or for specific species (dogs, cats, ornamental fishes). However, most participants positively rated the impact of their companion animals on their well-being during the pandemic, and qualitative data indicated that being a companion animal guardian was associated with a range of benefits in the context of COVID-19. These findings correspond with research conducted prior to the pandemic; there is a lack of convincing quantitative evidence to demonstrate differences in loneliness and well-being between companion animal guardians and non-guardians [22,55], yet qualitative studies show that people believe their companion animals positively influence their well-being [14,15,56,57].

### 4.1. Engagement with Companion Animals during the Pandemic

How people engage with their companion animals may influence the relationships between companion animal guardianship and loneliness and well-being [26,38]. The present study found evidence that spending more time than average talking to or petting dogs tended to be associated with more negative scores for loneliness and well-being, while the reverse was true for more positive scores. Causality cannot be established from the current data, but qualitative findings indicated that companionship, including physical and social interaction, was a benefit of keeping companion animals during the pandemic, particularly for more isolated individuals. This finding is corroborated by other research conducted during the pandemic [58]. More isolated individuals may therefore have experienced greater loneliness and poorer mental well-being during the pandemic, and subsequently spent more time engaging with their dogs as a substitute for interpersonal contact. Correspondingly, previous research has shown that among people with low levels of social support from humans, higher attachment to companion animals is associated with higher levels of loneliness [59]. However, contact with companion animals was an imperfect substitute for contact with other humans. Future research may wish to identify which aspects of interpersonal contact can and cannot be replicated by companion animals in order to identify which populations may benefit most from human–animal interactions.

The relationships between level of engagement with cats and well-being were less clear; more positive scores were associated with both greater than average and lower than average levels of engagement. A possible reason for this finding is that personality differences between dog and cat guardians influenced their experiences of the pandemic. Previous research has shown that self-identified ‘dog people’ tend to be more extraverted than ‘cat people’ [60]. While extraversion is usually associated with less loneliness and greater well-being, these protective effects may have been lost in the context of the pandemic due to restrictions on social interaction [61]. Due to their more extraverted nature, dog guardians may have had a greater desire for interpersonal contact which could not be satisfied through engagement with their companion animals. Cat guardians may have experienced a lesser need for social interaction, and so in some cases found that engagement with their companion animal was sufficient to alleviate the negative impacts of the pandemic. Further research is needed to examine how personality traits may interact with companion animal guardianship to influence the effects of social isolation.

Previous research has suggested that being a dog guardian may have greater benefits than the guardianship of other companion animals [62,63,64,65]. The current study found that primary or shared responsibility for a dog was associated with more positive outcomes for all dependent variables, suggesting that actively caring for a dog has benefits beyond the simple presence of a dog in the home. A probable explanation relates to the need to exercise dogs on a regular basis. Not only is there a known link between frequent physical activity and improved mental health [66], qualitative data indicated that dog walking had additional benefits, such as providing opportunities for social interaction and to spend time outdoors. Spending less time than average walking dogs was also associated with higher loneliness, possibly because participants had fewer opportunities for social interaction (although this finding is dependent on self-report data which were not verified with the use of pedometers or activity trackers). Previous research conducted both prior to and during the pandemic corroborates the finding that dog walking is associated with increased social connectedness [58,67,68,69], while numerous studies have demonstrated benefits associated with spending time in nature [70,71]. As having a greater level of responsibility for a dog presumably increases the likelihood of walking that dog, this would explain why more positive outcomes are associated with primary or shared responsibility of a dog, but not for cats or ornamental fishes.

### 4.2. Negative Aspects of Companion Animal Guardianship during the Pandemic

Both quantitative and qualitative data indicated that relatively few participants had experienced issues ensuring the welfare of their companion animals during the pandemic. Despite this finding, qualitative data suggested that proper care of companion animals was a significant source of worry for guardians during this time; this finding is corroborated by other research conducted during the pandemic [72,73]. Previous research has also indicated that people are willing to delay hospitalisation [74] or refuse access to support services [75] for the sake of their companion animals, and similar attitudes were present in the current study. Participants were often more concerned about the potential negative impact of COVID-19 on their companion animals than on themselves. For instance, while most participants had no concerns about transmitting SARS-CoV-2 either to or from their companion animals, a slightly larger proportion reported being concerned about transmitting the virus to their companion animals than the reverse; this finding is replicated in other research [73]. Therefore, the relationship between companion animal guardianship and well-being during the pandemic may have been confounded by the unique stresses associated with keeping companion animals.

A small number of individuals had very negative attitudes regarding the influence of their companion animals on their well-being during the pandemic. Although these individuals were very much in the minority, it is important to acknowledge their experiences, particularly as research in the HAI discipline is subject to self-selection biases; individuals are more likely to partake if they have positive attitudes towards companion animals [27]. As such, the current study findings should be interpreted with this population in mind, and future research should aim to blind study participants wherever possible to reduce the impact of these biases.

### 4.3. Ornamental Fishes and the Pandemic

A secondary aim of this research was to investigate the specific relationship between keeping ornamental fishes and loneliness and mental well-being during the pandemic. Quantitative analyses provided no evidence of an association between keeping ornamental fishes and scores on the standardised assessments; there was also no evidence that these relationships were influenced by the level of engagement between participants and their fishes. Very little research has examined the relationship between ornamental fish guardianship and loneliness and mental well-being [30], but these findings are in agreement with one study which examined the effects of adopting two goldfish on well-being among older adults; after six months no differences were found in loneliness, anxiety, leisure satisfaction, or happiness between the group who were given the goldfish and a no-treatment control [31].

Most participants rated ornamental fishes as having a positive impact on their well-being during the pandemic, but around a quarter appeared to feel indifferently and reported that their fishes had no effect. A likely explanation is that ornamental fishes are not viewed as companions in the same way as animals such as dogs and cats. Though some people who kept fishes felt they had bonded with their animals, many appreciated their home aquaria on a purely aesthetic level; watching fishes was an enjoyable and relaxing activity but provided little in the way of social or emotional support. Correspondingly, previous research found that many people who kept ornamental fishes associated them with benefits such as stress reduction, but 22% also viewed their home aquarium as ‘room decoration’ [76]. Physical touch was identified as an important aspect of the support provided by companion animals, a finding corroborated by other research conducted during the pandemic [58,67,72]. As fishes cannot interact with humans in a physical manner, it is therefore unsurprising that they were perceived as having a slightly less positive impact on the well-being of their guardians compared to other types of companion animals.

However, some aspects of fishkeeping were perceived to have positively impacted well-being during the pandemic. The need to provide ongoing care, irrespective of animal type, helped participants maintain a normal routine and made them feel needed. The relaxing effect of watching home aquaria was also commonly cited as a benefit of keeping ornamental fishes, though no association was found between time spent watching fishes each day and outcomes relating to relaxation, such as stress or anxiety. Current evidence regarding the relaxing effects of fish aquaria is inconclusive; although some studies have demonstrated results indicative of relaxation [37,77,78], others have failed to find evidence of an effect [79,80], and methodological issues make it difficult to draw firm conclusions [30]. Furthermore, most studies finding positive results have assessed outcomes immediately after viewing an aquarium, with a lack of evidence regarding how any transient benefits of watching a fish tank may translate into longer-term effects on well-being. As it is evident from this study and past research [32,76] that people believe watching fishes promotes relaxation, further research is needed to determine the specific circumstances under which this relaxation is induced, and how this may relate to overall well-being among ornamental fish guardians.

### 4.4. Strengths and Limitations

The mixed-methods design used in the current research provides a more complete understanding of the role that companion animals (and specifically, ornamental fishes) played in loneliness and mental well-being during the pandemic. Quantitative analysis is useful in summarising and comparing large quantities of data, but qualitative findings are needed to support the refinement of theory, and understand why interaction with companion animals may or may not influence well-being outcomes; despite this there is a lack of rigorous qualitative HAI research [28]. While cross-sectional data showed no association between companion animal guardianship and loneliness or well-being, qualitative findings demonstrated that companion animal guardians believe such effects exist. Identifying the specific aspects of keeping companion animals that were beneficial during the pandemic allows researchers to develop and test more nuanced theories regarding the importance of companion animals in the lives of their humans. Furthermore, the use of semi-structured interviews provided a deeper insight into the experiences of companion animal guardians than could be achieved through open-ended survey questions alone. While preferably qualitative data analysis would be conducted collaboratively by two authors, this was not possible due to time constraints. However, the final report was reviewed by a second author familiar with the data to ensure that the dataset was accurately and fully represented.

A cross-sectional design was used as the unprecedented nature of the pandemic precluded collection of baseline data. There were significant correlations between participants’ ratings of how their loneliness and well-being had changed during the pandemic and their scores on the standardised assessments, suggesting that to some degree these scores captured how participants had been affected by the pandemic. However, these scores likely also reflected participants’ levels of loneliness and mental well-being pre-pandemic. Some research has indicated that companion animal guardians may have poorer mental health than non-guardians [19,21], so it is possible that no associations were found between companion animal guardianship and loneliness and mental well-being in the current study, because existing differences between the groups masked any protective effects of companion animal guardianship. A UK-based study conducted during the first lockdown found that companion animal guardians experienced less deterioration in well-being and smaller increases in loneliness than those without companion animals, but these differences were very small, and the authors cautioned against claiming that companion animals strongly protected against the negative consequences of the pandemic [72].

Unlike other research conducted during the pandemic [58,67,72,81], the current study was not conducted under strict lockdown conditions. Consequently, participants will have experienced greater variation in COVID-19-related restrictions than in other research. This may explain why the statistical models explained relatively little variation in participants’ scores on the standardised assessments, despite accounting for a variety of demographic, social and lifestyle factors beyond companion animal guardianship. In particular, variation in employment status was reduced to a dichotomous variable, and it is possible that companion animal guardianship may have had differing influences for those whose employment was affected by the pandemic. Internet access was also required, which likely limited the number of older adults able to participate; as this population was at higher risk from COVID-19, different effects may have been identified if the sample contained more older adults. The study findings should therefore be interpreted in the context of the sample from which they were derived: predominantly white, female, residents in either the UK or USA, and quite highly educated relative to the general population.

### 4.5. Future Considerations

It has been argued that the ways individuals engage with their companion animals may influence the relationship between companion animal guardianship and well-being [26,38], and there was evidence to support this in the current study. Further research is needed to consider which elements of HAI are most important for well-being, so they may be accounted for in data analysis; qualitative findings from this study and other research may be used to refine and test existing HAI theories to identify these elements.

While the collection of pre-pandemic data was not possible for the current study, it may be possible to utilise existing data to compare pre- and post-pandemic effects of companion animal guardianship. Previously, data from large-scale longitudinal studies have been used to examine the impact of companion animal guardianship on well-being [82]. Where such studies have collected data pre-and post-pandemic, it may be possible to examine any longer-term influences of companion animal guardianship during the pandemic and beyond.

Finally, further research is needed regarding the specific impact of ornamental fishes on well-being within the context of companion animal guardianship. To date, most research has focused on the shorter-term impacts of fish aquaria in a clinical or health care context [30], and while qualitative data from this and other research [32] have suggested that watching fish aquaria can induce relaxation, there is no quantitative data to support this within the home environment. It is also unclear whether fish guardianship translates to longer-term effects on well-being, and whether certain populations experience greater or lesser benefits from their home aquaria. Future research should aim to address these gaps in the evidence.

## 5. Conclusions

In conclusion, there was no quantitative evidence that being a companion animal guardian was associated with less loneliness and greater mental well-being during the COVID-19 pandemic, nor was this influenced by specific companion animal types (dogs, cats, ornamental fishes). There was some evidence that a greater level of engagement with dogs, and to a lesser extent cats, was associated with higher loneliness and poorer mental well-being, which may indicate that these individuals experienced higher levels of social isolation. Further research is needed to investigate this theory. Qualitative data indicated that companion animal guardians had experienced a range of benefits during the pandemic; these insights may be useful in refining theories regarding the impact of companion animals on human well-being. Negative aspects of companion animal guardianship during the pandemic typically related to animal welfare concerns, but a small number of participants had very negative experiences regarding the impact of their companion animals on their well-being. These experiences warrant further consideration, as they may shed light on conflicting findings within HAI research. Findings regarding ornamental fish guardians generally reflected those of companion animals more broadly, though there was evidence that a larger proportion held indifferent views regarding the impact of their companion animals on their well-being, and level of engagement with ornamental fishes was not associated with loneliness or well-being. More research is needed to understand the impact of ornamental fishes within the context of companion animal guardianship.

## Figures and Tables

**Table 1:** Description of survey questions by section of survey.

Section of Survey	Description
Section 1: Demographic details	Closed questions: age group, gender, ethnicity, highest level of education, marital status, country of residence, and number of adults and children living in the household.
Section 2: Social and lifestyle behaviours during the pandemic	Closed questions regarding the level of social interaction experienced during the pandemic; frequency of communication with friends and family outside the household via written methods (e.g., text messaging, email), verbal methods (e.g., phone or video calls) or face to face, and frequency of having left the home to exercise or complete essential errands (responses from 1 = “Never” to 7 = “More than once a day”); employment status during the pandemic; period of self-isolation greater than one week (yes/no).
Section 3: Companion animal guardianship and level of engagement	Mix of closed and open questions. Participants who kept companion animals indicated which types (dogs, cats, fish, small mammals, exotic animals, birds, and/or other), and for each type: The number of individuals (plus number of tanks/ponds for fishes). If they were the primary caregiver(s). Where the animal lived (inside the home/outside). Where the animal slept (dogs and cats only). Whether interaction with that animal type had been affected by the pandemic (open-ended question). The perceived influence of that animal type on their well-being during the pandemic (1 = “Extremely negative” to 7 = “Extremely positive”). Level of engagement, i.e., the amount of time spent undertaking different behaviours relating to that companion animal type on a typical day during the pandemic (1 = “None” to 8 = “More than four hours”); some behaviours were measured for all animal types (e.g., time spent feeding/talking to animals), some were species-specific (e.g., time spent walking dogs/conducting tank or pond maintenance). Level of engagement was measured rather than human–animal bond, as it is unclear how the latter applies within the fish guardianship dynamic. Additional questions regarding the general experience of companion animal guardianship during the pandemic included: Problems accessing supplies or veterinary treatment (yes/no). Other pandemic-related issues regarding care of companion animals. Level of concern about contracting/giving SARS-CoV-2 from/to companion animals (1 = “Not at all concerned” to 4 = “Extremely concerned”). Any new animals purchased/adopted during/because of the pandemic.
Section 4: Well-being assessments	Series of four validated scales to measure loneliness and well-being: The short-form UCLA Loneliness Scale (UCLA-LS) [41] measured overall loneliness. Thinking about their life “at the moment”, participants gave responses to three items (e.g., “How often do you feel that you lack companionship?”) on a 3-point scale from 1 = “Hardly ever” to 3 = “Often”. Scores were calculated by summing responses (min = 3, max = 9). This scale is commonly used to measure loneliness in HAI research [22], and has satisfactory levels of reliability and validity [41]. The De Jong Gierveld Loneliness Scale is considered a reliable and valid instrument to measure social, emotional and overall loneliness [42]. Responses to six items (e.g., “I miss having people around me”) could be “yes”, “more or less” or “no”. Participants responded with regard to how they were currently feeling. One point was given for items 1–3 if the response was "yes" or "more or less", and for items 4–6 if the response was "no" or "more or less". Emotional loneliness was calculated by summing items 1–3, social loneliness by summing items 4–6 (min = 0, max = 3 for each), and overall loneliness by summing all items (min = 0, max = 6). The Warwick–Edinburgh Mental Well-Being Scale (WEMWBS) [43] measured mental well-being. Participants indicated how much 14 statements (e.g., “I’ve been feeling optimistic about the future”) had applied to them over the past two weeks using a 5-point scale (1 = “None of the time” to 5 = “All of the time”). Scores were calculated by summing responses to all items (min = 14, max = 70). The WEMWBS is used extensively in psychological research and is considered psychometrically sound [43]. The long version was used as it covers both psychological functioning and positive affect, while the short version covers only psychological functioning. The short-form of the Depression Anxiety Stress Scales (DASS-21) [44] required participants to respond to 21 statements (e.g., “I found it hard to wind down”) using a scale from 0 = “Did not apply to me at all” to 3 = “Applied to me very much” to indicate how much each statement had applied to them over the past two weeks. The timescale for the DASS-21 is usually the past week but was altered to match the WEMWBS. A total score for each construct was calculated by summing responses to all items from the relevant scales (min = 0, max = 21 per subscale). The DASS-21 has high internal consistency and discriminant validity [45].
Section 5: Rating scales for change in well-being during the pandemic	Participants indicated how anxious, low or depressed, stressed, and lonely they had felt during the pandemic, on a scale from 1 = “Much less than normal” to 7 = “Much more than normal”. Overall well-being during the pandemic was rated on a scale from 1 = “Much worse than usual” to 7 = “Much better than usual”. These items were used to support the understanding of whether any relationships identified between companion animal guardianship and well-being related to the influence of companion animals during the pandemic or are tapping into existing differences in the populations.
Section 6: Open-ended questions	Two items allowed participants to describe in their own words how they had supported their mental well-being during the pandemic, and the specific contribution of their companion animals (if they had any).

**Table 2:** Survey participant characteristics by companion animal guardianship status.

	All Companion Animal Guardians (*n* = 1005)	Non-Companion Animal Guardians (*n* = 154)	Total (*n* = 1159)
**Age**			
18–34	364 (36%)	74 (48%)	438 (38%)
35–50	353 (35%)	41 (27%)	394 (34%)
51–69	254 (25%)	27 (18%)	281 (24%)
70+	32 (3%)	12 (8%)	44 (4%)
Prefer not to say	2 (<1%)	0	2 (<1%)
**Gender**			
Female	839 (83%)	111 (72%)	950 (82%)
Male	154 (15%)	41 (27%)	195 (17%)
Other	4 (<1%)	2 (1%)	6 (1%)
Prefer not to say	8 (1%)	0	8 (1%)
**Ethnicity**			
White	920 (92%)	141 (92%)	1061 (92%)
Asian	18 (2%)	5 (3%)	23 (2%)
Black or African American	5 (<1%)	1 (1%)	6 (1%)
Hispanic or Latino	32 (3%)	3 (2%)	35 (3%)
Two or more	12 (1%)	1 (1%)	13 (1%)
Prefer not to say/unclear	18 (2%)	3 (2%)	21 (2%)
**Education**			
High school or below	135 (13%)	15 (10%)	150 (13%)
Undergraduate	415 (41%)	47 (31%)	462 (40%)
Masters	270 (27%)	57 (37%)	327 (28%)
Doctorate	172 (17%)	35 (23%)	207 (18%)
Prefer not to say/don’t know	13 (1%)	0	13 (1%)
**Marital**			
Married/living with partner	620 (62%)	77 (50%)	697 (60%)
Partner, not living together	57 (6%)	14 (9%)	71 (6%)
Single, separated, divorced or widowed	320 (32%)	61 (40%)	381 (33%)
Prefer not to say/unclear	8 (1%)	2 (1%)	10 (1%)
**Live with children**			
Yes	244 (24%)	38 (25%)	282 (24%)
No	761 (76%)	116 (75%)	877 (76%)
**Country**			
United Kingdom	473 (47%)	113 (73%)	586 (51%)
United States	351 (35%)	24 (16%)	375 (32%)
Other	179 (18%)	16 (10%)	195 (17%)
Prefer not to say	2 (<1%)	1 (1%)	3 (<1%)
**Frequency of written communication**			
Less than an average amount	390 (39%)	64 (42%)	454 (39%)
An average amount	615 (61%)	90 (58%)	705 (61%)
**Frequency of verbal communication**			
Less than an average amount	305 (30%)	31 (20%)	336 (29%)
An average amount	404 (40%)	61 (40%)	465 (40%)
More than an average amount	296 (29%)	62 (40%)	358 (31%)
**Frequency of face-to-face communication**			
Less than an average amount	330 (33%)	48 (31%)	378 (33%)
An average amount	258 (26%)	38 (25%)	296 (26%)
More than an average amount	416 (41%)	67 (44%)	483 (42%)
Prefer not to say	1 (<1%)	1 (1%)	2 (<1%)
**Frequency of exercise outside the home**			
Less than an average amount	323 (32%)	44 (29%)	367 (32%)
An average amount	233 (23%)	50 (32%)	283 (24%)
More than an average amount	445 (44%)	60 (39%)	505 (44%)
Prefer not to say	4 (<1%)	0	4 (<1%)
**Frequency of essential errands**			
Less than an average amount	291 (29%)	41 (27%)	332 (29%)
An average amount	387 (39%)	69 (45%)	456 (39%)
More than an average amount	327 (33%)	44 (29%)	371 (32%)
**Working**			
Yes	756 (75%)	122 (79%)	878 (76%)
No	241 (24%)	32 (21%)	273 (24%)
Prefer not to say	8 (1%)	0	8 (1%)
**Period of isolation greater than 1 week**			
Yes	305 (30%)	31 (20%)	336 (29%)
No	697 (69%)	123 (80%)	820 (71%)
Prefer not to say	3 (<1%)	0	3 (<1%)

Note: ‘Other’ genders were recorded as missing data for analyses; examples of unclear responses include “human being” and “English” for ethnicity, and “boyfriend” and “engaged” for marital status.

**Table 3:** Example quotations for “Companion animals as providers of social support”.

Subtheme	Example Quotations
It meant never being alone	“They are company, comfort, a living presence in my empty home” (Survey, Participant 677) “…my husband his work is outside of the home, so during lockdown he had to be at home…when he left it was a big change because we were like three months, you know, we were on top of each other in a one bedroom flat and it was really, really nice because you know I’d get on with work and he’d be doing things around the house and yeah he was just there, but then when he started going back to work I was like “oh my gosh” you know, and then I’m not going back to work, I’m here at home still so I think at that point having the cats and dog became even more important” (Interview, Participant 21) “…having her for company in a time when I am very isolated has been not a perfect substitute for human interaction but it has been something…” (Survey, Participant 318)
A substitute for human talk and touch	“As I’ve had less face to face contact with friends and family my dog’s company has been exceptionally important. I think I would have felt very alone otherwise. I normally have a hug with friends/family, so it’s been good to have my dog curl up next to me for a cuddle.” (Survey, Participant 51) “They have been company for me and the only other beings I could have any physical contact with for three full months. They’re very loving and sweet, but I’ve still felt the lack of a human hug too—dog snuggles are lovely but it’s also not the same as being held.” (Survey, Participant 222) “…even though they don’t answer me back, to be able to speak with them in such a way that it’s humanising, yes it’s anthropomorphising them but that’s part of it, that yes you can talk with them and they do seem to respond and you know, a dog cocks his head when you’re talking to him and it’s because he’s confused but it’s still—you know he’s listening…” (Interview, Participant 14)
Bridging interpersonal connections during social distancing	“When I have taken my dogs out it has sometimes been because of them that strangers might at least say hello, this may have been my only interaction with a human some days” (Survey, Participant 96) “…it’s been really nice to sort of see everyone on zoom calls with their respectively pets and random cats and dogs wandering into frame, so I think that’s been quite nice to see as well, it kind of builds a like ‘oh I didn’t know you had a dog’ or ‘what type of dog do you have’, so that’s been nice…” (Interview, Participant 19) “…I’ve really enjoyed learning new things about the fish or keeping the fish etc. I’ve also enjoyed—I use Instagram and I share pictures [of aquaria] and things on there, which has been cool because I’ve met and been speaking to new people…” (Interview, Participant 10) “…my son [son’s name] wants me to send photos of the dogs and things like that so, so yeah I think because maybe people have got more time on their hands—other people in the family—they’ve wanted to do more with them…” (Interview, Participant 24)

**Table 4:** Example quotations for “Companion animals as providers of purpose and perspective”.

Subtheme	Example Quotations
They help maintain a normal routine	“…one thing with the fish particularly, because they need quite a lot of ongoing care, you know they need regular water changes, you know, all the looking after of the water, you can’t sort of think ‘oh I really can’t be bothered this week’ you have to do it, and having that routine has actually been quite a good thing—everybody laughs about you don’t know what day of the week it is let alone what month of the year it is, because every day’s just the same at the moment, and actually having that regular routine with the fish has been a good thing actually…” (Interview, Participant 17) “They are normal. They act normal. The routine is normal. They seem to like that I am home more. At home, things are normal. This makes it easier to cope with things not being normal outside the home. And makes it easier to stay home.” (Survey, Participant 659) “Having a dog has definitely helped because I have walked every single day of lockdown so it’s a great excuse for exercise, which also gave me the opportunity to get out and explore what’s on my doorstep, so I’ve discovered new things about where I live. The feeling of getting away from the four walls when we could only exercise once a day was a real mental health lifesaver…” (Survey, Participant 348)
Something good to focus on	“…you feel out of control, you don’t know who’s going to get sick, when and how badly, there’s a lot of fear and those little moments of escaping that to deal with a companion [animal] is invaluable…” (Interview, Participant 23) “…it’s so nice on an evening or something if I’m feeding them, they’ll come running in and playing and they’re really cute and make me laugh, they’re better than the television…” (Interview, Participant 5) “The fish tank has provided relaxation as you can sit and stare at it for a long time and it keeps interest.” (Survey, Participant 462)
A reason to keep going	“I have struggled with feeling useless due to being furloughed but having the dog to feed/walk/train has given my days structure and purpose” (Survey, Participant 668) “…the responsibility of making sure they are okay is motivating to keep going and look after myself also and, in the extreme times, it’s an important reason to stay alive…” (Survey, Participant 706) “Overall the fish have had a positive influence during this time. But there has been anxiety as my fish needed a bigger tank. Impossible to get a tank during lockdown. I had issues buying seafood for my fish as panic buyers bought it all. I had trouble with buying essential items locally. When I thought I had COVID and was told to go into hospital I had no one to look after my fish. So this influenced my decision to stay in hospital.” (Survey, Participant 469) “…I’ve got a cupboard absolutely stashed with the kind of food that I think she’s probably going to like and it’s ok, just because if suddenly I can’t go to the shops “oh my gosh” so—and I’m probably more so on her behalf because for myself, ok I’ll just have plain pasta and I’m quite ok with that but I feel more responsible for her well-being and I want to make sure that she’s got everything she needs…” (Interview, Participant 20)

## Data Availability

The quantitative data presented in this study are available on request from the corresponding author. These data are not publicly available because permission to share data publicly was not obtained from participants as part of the consent process. To ensure participant confidentiality, qualitative data cannot be shared outside the research team.

## Supplementary Materials

The following are available online at https://www.mdpi.com/article/10.3390/ani11082349/s1, Table S1: Survey participant characteristics by type of companion animal; Table S2: Results of robust hierarchical multiple regression analyses; Table S3: Results of robust hierarchical regression analyses for level of engagement with companion animals.
