# Supplementary material for: Companion Animal Type and Level of Engagement Matter: A Mixed-Methods Study Examining Links between Companion Animal Guardianship, Loneliness and Well-Being during the COVID-19 Pandemic
Source: Animals (Basel). 2021 Aug 9;11(8):2349. doi: 10.3390/ani11082349 (PMC8388758; doi:10.3390/ani11082349)
Supplement: Supplementary file 1 [file animals-11-02349-s001.zip › animals-1258426-supplementary.pdf]

Table S1. Survey participant characteristics by type of companion animal.

	Dog guardians (n =661)	Cat guardians (n =469)	Fish guardians (n =218)	Other companion animal guardians (n =226)	All companion animal guardians (n =1005)	Non-companion animal guardians (n =154)	Total (n =1159)
**Age**							
18-34	214 (32%)	172 (37%)	98 (45%)	105 (46%)	364 (36%)	74 (48%)	438 (38%)
35-50	241 (36%)	169 (36%)	77 (35%)	75 (33%)	353 (35%)	41 (27%)	394 (34%)
51-69	185 (28%)	112 (24%)	39 (18%)	43 (19%)	254 (25%)	27 (18%)	281 (24%)
70+	20 (3%)	16 (3%)	3 (1%)	3 (1%)	32 (3%)	12 (8%)	44 (4%)
Prefer not to say	1 (<1%)	0	1 (<1%)	0	2 (<1%)	0	2 (<1%)
**Gender**							
Female	554 (84%)	404 (86%)	157 (72%)	196 (87%)	839 (83%)	111 (72%)	950 (82%)
Male	102 (15%)	57 (12%)	59 (27%)	28 (12%)	154 (15%)	41 (27%)	195 (17%)
Other	2 (<1%)	3 (1%)	0	1 (<1%)	4 (<1%)	2 (1%)	6 (1%)
Prefer not to say	3 (<1%)	5 (1%)	2 (1%)	1 (<1%)	8 (1%)	0	8 (1%)
**Ethnicity**							
White	604 (91%)	429 (91%)	205 (94%)	219 (97%)	920 (92%)	141 (92%)	1061 (92%)
Asian	11 (2%)	10 (2%)	4 (2%)	2 (1%)	18 (2%)	5 (3%)	23 (2%)
Black or African American	4 (1%)	3 (1%)	0	1 (<1%)	5 (<1%)	1 (1%)	6 (1%)
Hispanic or Latino	24 (4%)	15 (3%)	0	0	32 (3%)	3 (2%)	35 (3%)
Two or more	8 (1%)	4 (1%)	3 (1%)	2 (1%)	12 (1%)	1 (1%)	13 (1%)
Prefer not to say/unclear	10 (2%)	8 (2%)	6 (3%)	2 (1%)	18 (2%)	3 (2%)	21 (2%)
**Education**							
High school or below	91 (14%)	64 (14%)	42 (19%)	43 (19%)	135 (13%)	15 (10%)	150 (13%)
Undergraduate	269 (41%)	198 (42%)	100 (46%)	99 (44%)	415 (41%)	47 (31%)	462 (40%)
Masters	183 (28%)	127 (27%)	49 (22%)	42 (19%)	270 (27%)	57 (37%)	327 (28%)
Doctorate	111 (17%)	75 (16%)	21 (10%)	38 (17%)	172 (17%)	35 (23%)	207 (18%)
Prefer not to say/don’t know	7 (1%)	5 (1%)	6 (3%)	4 (2%)	13 (1%)	0	13 (1%)
**Marital**							
Married/living with partner	426 (64%)	276 (59%)	145 (67%)	137 (61%)	620 (62%)	77 (50%)	697 (60%)
Partner, not living together	26 (4%)	30 (6%)	20 (9%)	16 (7%)	57 (6%)	14 (9%)	71 (6%)
Single, separated, divorced or widowed	206 (31%)	156 (33%)	51 (23%)	72 (32%)	320 (32%)	61 (40%)	381 (33%)
Prefer not to say/unclear	3 (<1%)	7 (1%)	2 (1%)	1 (<1%)	8 (1%)	2 (1%)	10 (1%)
**Live with children**							
Yes	175 (26%)	125 (27%)	80 (37%)	72 (32%)	244 (24%)	38 (25%)	282 (24%)
No	486 (74%)	344 (73%)	138 (63%)	154 (68%)	761 (76%)	116 (75%)	877 (76%)
**Country**							
United Kingdom	277 (42%)	216 (46%)	139 (64%)	139 (62%)	473 (47%)	113 (73%)	586 (51%)

United States	244 (37%)	174 (37%)	52 (24%)	60 (27%)	351 (35%)	24 (16%)	375 (32%)
Other	139 (21%)	78 (17%)	27 (12%)	27 (12%)	179 (18%)	16 (10%)	195 (17%)
Prefer not to say	1 (<1%)	1 (<1%)	0	0	2 (<1%)	1 (1%)	3 (<1%)
**Frequency of written communication**							
Less than an average amount	261 (39%)	185 (39%)	81 (37%)	92 (41%)	390 (39%)	64 (42%)	454 (39%)
An average amount	400 (61%)	284 (61%)	137 (63%)	134 (59%)	615 (61%)	90 (58%)	705 (61%)
**Frequency of verbal communication**							
Less than an average amount	204 (31%)	148 (32%)	69 (32%)	78 (35%)	305 (30%)	31 (20%)	336 (29%)
An average amount	249 (38%)	198 (42%)	92 (42%)	91 (40%)	404 (40%)	61 (40%)	465 (40%)
More than an average amount	208 (31%)	123 (26%)	57 (26%)	57 (25%)	296 (29%)	62 (40%)	358 (31%)
**Frequency of face-to-face communication**							
Less than an average amount	214 (32%)	161 (34%)	76 (35%)	65 (29%)	330 (33%)	48 (31%)	378 (33%)
An average amount	163 (25%)	119 (25%)	51 (23%)	58 (26%)	258 (26%)	38 (25%)	296 (26%)
More than an average amount	284 (43%)	188 (40%)	91 (42%)	103 (46%)	416 (41%)	67 (44%)	483 (42%)
Prefer not to say	0	1 (<1%)	0	0	1 (<1%)	1 (1%)	2 (<1%)
**Frequency of exercise outside the home**							
Less than an average amount	195 (30%)	171 (36%)	77 (35%)	67 (30%)	323 (32%)	44 (29%)	367 (32%)
An average amount	119 (18%)	121 (26%)	57 (26%)	53 (23%)	233 (23%)	50 (32%)	283 (24%)
More than an average amount	345 (52%)	175 (37%)	84 (39%)	106 (47%)	445 (44%)	60 (39%)	505 (44%)
Prefer not to say	2 (<1%)	2 (<1%)	0	0	4 (<1%)	0	4 (<1%)
**Frequency of essential errands**							
Less than an average amount	190 (29%)	139 (30%)	63 (29%)	66 (29%)	291 (29%)	41 (27%)	332 (29%)
An average amount	260 (39%)	188 (40%)	72 (33%)	81 (36%)	387 (39%)	69 (45%)	456 (39%)
More than an average amount	211 (32%)	142 (30%)	83 (38%)	79 (35%)	327 (33%)	44 (29%)	371 (32%)
**Working**							
Yes	494 (75%)	356 (76%)	147 (67%)	167 (74%)	756 (75%)	122 (79%)	878 (76%)
No	163 (25%)	110 (23%)	69 (32%)	58 (26%)	241 (24%)	32 (21%)	273 (24%)
Prefer not to say	4 (1%)	3 (1%)	2 (1%)	1 (<1%)	8 (1%)	0	8 (1%)
**Period of isolation greater than 1 week**							
Yes	189 (29%)	149 (32%)	72 (33%)	64 (28%)	305 (30%)	31 (20%)	336 (29%)
No	471 (71%)	319 (68%)	145 (67%)	161 (71%)	697 (69%)	123 (80%)	820 (71%)
Prefer not to say	1 (<1%)	1 (<1%)	1 (<1%)	1 (<1%)	3 (<1%)	0	3 (<1%)

Note: ‘Other’ genders were recorded as missing data for analyses; examples of unclear responses include “human being” and “English” for ethnicity, and “boyfriend” and “engaged” for marital status.

Table S2. Results of robust hierarchical multiple regression analyses.

	Mental well-being (n = 1031)				UCLA Loneliness Scale (n = 1074)				Depression (n = 1046)			
	$\hat{\beta}$	SE	t	*p*	$\hat{\beta}$	SE	t	*p*	$\hat{\beta}$	SE	t	*p*
**Step 1**	R^2 = 0.072<br>χ^2 (12) = 74.94, p <0.001				R^2 = 0.129<br>χ^2 (12) = 150.85, p <0.001				R^2 = 0.098<br>χ^2 (12) = 114.2, p <0.001			
Intercept	39.28	1.06	37.05	<0.001	6.23	0.19	33.53	<0.001	14.56	0.84	17.30	<0.001
**Age** (0=18-34 years)												
35-50 years	**1.53**	**0.75**	**2.04**	**0.042**	**-0.49**	**0.13**	**-3.66**	**<0.001**	**-2.23**	**0.60**	**-3.70**	**<0.001**
51-69 years	**3.08**	**0.81**	**3.80**	**<0.001**	**-0.47**	**0.15**	**-3.19**	**0.001**	**-2.95**	**0.66**	**-4.50**	**<0.001**
70+ years	**6.69**	**1.63**	**4.12**	**<0.001**	**-0.83**	**0.29**	**-2.86**	**0.004**	**-4.46**	**1.29**	**-3.46**	**0.001**
**Gender** (0=Female)												
Male	1.44	0.81	1.79	0.074	-0.22	0.14	-1.56	0.119	**-1.30**	**0.65**	**-2.00**	**0.046**
**Highest level of education** (0=School or below)												
Undergraduate	**2.35**	**0.99**	**2.38**	**0.017**	-0.19	0.17	-1.10	0.272	-1.24	0.78	-1.58	0.114
Masters	**2.59**	**1.03**	**2.51**	**0.012**	-0.01	0.18	-0.08	0.939	-1.43	0.82	-1.74	0.082
Doctorate	**3.01**	**1.12**	**2.70**	**0.007**	-0.18	0.20	-0.91	0.362	**-2.10**	**0.89**	**-2.36**	**0.019**
**Marital status** (0=Single, separated, divorced, widowed)												
Partnered, not living together	2.46	1.31	1.88	0.060	**-0.68**	**0.24**	**-2.85**	**0.004**	0.51	1.06	0.48	0.634
Married or living with partner	**2.76**	**0.67**	**4.11**	**<0.001**	**-1.18**	**0.12**	**-9.81**	**<0.001**	**-1.84**	**0.54**	**-3.38**	**0.001**
**Live with children** (0=No)												
Yes	1.22	0.76	1.60	0.110	-0.13	0.14	-0.99	0.320	**-1.90**	**0.61**	**-3.10**	**0.002**
**Country** (0=United Kingdom)												
United States	0.93	0.71	1.31	0.192	<0.01	0.13	-0.04	0.969	**-1.88**	**0.57**	**-3.30**	**0.001**
Other	**2.15**	**0.86**	**2.49**	**0.013**	-0.09	0.15	-0.56	0.576	**-2.40**	**0.69**	**-3.46**	**0.001**
	Mental well-being (n = 1031)				UCLA Loneliness Scale (n = 1074)				Depression (n = 1046)			
	$\hat{\beta}$	SE	t	*p*	$\hat{\beta}$	SE	t	*p*	$\hat{\beta}$	SE	t	*p*
**Step 2**	ΔR^2 = 0.034<br>χ^2 (8) = 37.54, p <0.001				ΔR^2 = 0.036<br>χ^2 (8) = 42.54, p <0.001				ΔR^2 = 0.031<br>χ^2 (8) = 37.42, p <0.001			
Intercept	37.89	1.44	26.24	<0.001	6.42	0.26	25.14	<0.001	16.50	1.17	14.14	<0.001
**Age** (0=18-34 years)												
35-50 years	1.04	0.75	1.40	0.163	**-0.42**	**0.13**	**-3.17**	**0.002**	**-1.88**	**0.61**	**-3.11**	**0.002**
51-69 years	**3.06**	**0.81**	**3.75**	**<0.001**	**-0.42**	**0.15**	**-2.87**	**0.004**	**-2.93**	**0.66**	**-4.42**	**<0.001**
70+ years	**7.58**	**1.71**	**4.44**	**<0.001**	**-0.85**	**0.30**	**-2.80**	**0.005**	**-5.66**	**1.37**	**-4.15**	**<0.001**
**Gender** (0=Female)												
Male	1.18	0.80	1.48	0.140	-0.22	0.14	-1.57	0.118	-1.10	0.65	-1.70	0.089
**Highest level of education** (0=School or below)												

Undergraduate	1.76	0.98	1.80	0.073	-0.17	0.17	-0.97	0.334	-0.82	0.78	-1.05	0.293
Masters	**2.07**	**1.03**	**2.01**	**0.045**	<0.01	0.18	0.02	0.981	-1.05	0.83	-1.26	0.208
Doctorate	2.08	1.13	1.83	0.067	-0.14	0.20	-0.70	0.487	-1.39	0.91	-1.53	0.128
**Marital status** (0=Single, separated, divorced, widowed)												
Partnered, not living together	2.50	1.30	1.93	0.054	**-0.60**	**0.24**	**-2.56**	**0.011**	0.64	1.06	0.61	0.544
Married or living with partner	**2.72**	**0.67**	**4.06**	**<0.001**	**-1.17**	**0.12**	**-9.77**	**<0.001**	**-1.72**	**0.55**	**-3.14**	**0.002**
**Live with children** (0=No)												
Yes	1.18	0.75	1.57	0.116	-0.11	0.13	-0.84	0.404	**-1.88**	**0.61**	**-3.08**	**0.002**
**Country** (0=United Kingdom)												
United States	**1.43**	**0.73**	**1.97**	**0.049**	-0.11	0.13	-0.83	0.407	**-2.38**	**0.59**	**-4.02**	**<0.001**
Other	**2.36**	**0.87**	**2.71**	**0.007**	-0.20	0.15	-1.27	0.203	**-2.63**	**0.71**	**-3.71**	**<0.001**
**Communicated via verbal means** (0=Average)												
Less than average	**-1.80**	**0.73**	**-2.45**	**0.014**	0.17	0.13	1.30	0.195	0.97	0.59	1.63	0.104
More than average	-0.14	0.71	-0.19	0.847	0.15	0.13	1.14	0.254	0.13	0.58	0.22	0.824
**Communicated face-to-face** (0=Average)												
Less than average	-0.85	0.78	-1.09	0.275	0.08	0.14	0.59	0.553	-0.07	0.64	-0.11	0.910
More than average	0.42	0.75	0.56	0.574	**-0.29**	**0.14**	**-2.15**	**0.032**	-0.62	0.62	-1.01	0.312
**Left the house to exercise** (0=Average)												
Less than average	0.33	0.82	0.41	0.685	-0.28	0.15	-1.90	0.058	-0.32	0.67	-0.47	0.637
More than average	**2.64**	**0.74**	**3.57**	**<0.001**	**-0.39**	**0.13**	**-2.94**	**0.003**	**-2.26**	**0.60**	**-3.76**	**<0.001**
**Working during pandemic** (0=No)												
Yes	**1.91**	**0.79**	**2.42**	**0.016**	-0.11	0.14	-0.79	0.432	**-1.87**	**0.64**	**-2.92**	**0.004**
**Period of isolation >1 week** (0=No)												
Yes	-0.53	0.68	-0.79	0.431	**0.49**	**0.12**	**4.08**	**<0.001**	0.93	0.55	1.68	0.093
	Mental well-being (n = 1031)				UCLA Loneliness Scale (n = 1074)				Depression (n = 1046)			
	$\hat{\beta}$	SE	t	*p*	$\hat{\beta}$	SE	t	*p*	$\hat{\beta}$	SE	t	*p*
**Step 3**	$\Delta R^2 = 0.008$ $\chi^2(5) = 8.52$, $p = 0.130$				$\Delta R^2 = 0.001$ $\chi^2(5) = 1.40$, $p = 0.925$				$\Delta R^2 = 0.008$ $\chi^2(5) = 8.29$, $p = 0.141$			
Intercept	38.82	1.63	23.77	<0.001	6.43	0.29	22.28	<0.001	15.59	1.32	11.83	<0.001
**Age** (0=18-34 years)												
35-50 years	1.09	0.75	1.45	0.148	**-0.42**	**0.13**	**-3.10**	**0.002**	**-1.94**	**0.61**	**-3.19**	**0.001**
51-69 years	**3.15**	**0.82**	**3.83**	**<0.001**	**-0.40**	**0.15**	**-2.74**	**0.006**	**-3.03**	**0.67**	**-4.54**	**<0.001**
70+ years	**7.30**	**1.73**	**4.23**	**<0.001**	**-0.84**	**0.31**	**-2.74**	**0.006**	**-5.34**	**1.38**	**-3.88**	**<0.001**
**Gender** (0=Female)												
Male	0.90	0.82	1.10	0.273	-0.24	0.15	-1.67	0.095	-0.91	0.66	-1.37	0.171
**Highest level of education** (0=School or below)												
Undergraduate	1.71	0.98	1.74	0.082	-0.17	0.17	-0.96	0.337	-0.67	0.79	-0.85	0.395
Masters	1.95	1.04	1.87	0.062	0.01	0.18	0.05	0.959	-0.81	0.84	-0.97	0.334
Doctorate	2.02	1.15	1.77	0.078	-0.13	0.20	-0.66	0.508	-1.14	0.92	-1.24	0.217

**Marital status** (0=Single, separated, divorced, widowed)												
Partnered, not living together	2.22	1.30	1.70	0.089	**-0.62**	**0.24**	**-2.63**	**0.009**	0.77	1.06	0.73	0.467
Married or living with partner	**2.84**	**0.68**	**4.21**	**<0.001**	**-1.16**	**0.12**	**-9.62**	**<0.001**	**-1.84**	**0.55**	**-3.34**	**0.001**
**Live with children** (0=No)												
Yes	1.13	0.77	1.46	0.144	-0.13	0.14	-0.94	0.349	**-2.03**	**0.62**	**-3.26**	**0.001**
**Country** (0=United Kingdom)												
United States	**1.88**	**0.75**	**2.50**	**0.013**	-0.08	0.13	-0.60	0.547	**-2.72**	**0.61**	**-4.47**	**<0.001**
Other	**2.89**	**0.90**	**3.22**	**0.001**	-0.16	0.16	-0.99	0.325	**-3.01**	**0.73**	**-4.13**	**<0.001**
**Communicated via verbal means** (0=Average)												
Less than average	**-1.62**	**0.74**	**-2.20**	**0.028**	0.18	0.13	1.35	0.177	0.86	0.60	1.45	0.148
More than average	-0.09	0.72	-0.13	0.897	0.16	0.13	1.21	0.227	0.18	0.58	0.31	0.759
**Communicated face-to-face** (0=Average)												
Less than average	-0.91	0.78	-1.17	0.244	0.08	0.14	0.56	0.578	-0.06	0.64	-0.10	0.920
More than average	0.46	0.76	0.61	0.544	**-0.29**	**0.14**	**-2.15**	**0.031**	-0.71	0.62	-1.15	0.249
**Left the house to exercise** (0=Average)												
Less than average	0.39	0.82	0.47	0.639	-0.28	0.15	-1.88	0.060	-0.43	0.67	-0.64	0.525
More than average	**3.10**	**0.76**	**4.06**	**<0.001**	**-0.36**	**0.14**	**-2.66**	**0.008**	**-2.60**	**0.62**	**-4.21**	**<0.001**
**Working during pandemic** (0=No)												
Yes	**1.90**	**0.79**	**2.40**	**0.017**	-0.10	0.14	-0.72	0.474	**-1.82**	**0.64**	**-2.83**	**0.005**
**Period of isolation >1 week** (0=No)												
Yes	-0.57	0.68	-0.84	0.402	**0.49**	**0.12**	**4.02**	**<0.001**	0.95	0.55	1.72	0.087
**Companion animal guardian** (0=No)												
Yes	-1.20	1.19	-1.01	0.315	-0.06	0.21	-0.29	0.771	0.07	0.97	0.07	0.944
**Dog guardian** (0=No)												
Yes	-1.08	0.80	-1.35	0.179	-0.07	0.14	-0.47	0.640	**1.38**	**0.65**	**2.13**	**0.034**
**Cat guardian** (0=No)												
Yes	0.44	0.73	0.60	0.549	0.02	0.13	0.19	0.852	0.55	0.59	0.93	0.355
**Fish guardian** (0=No)												
Yes	1.03	0.82	1.25	0.212	0.12	0.15	0.79	0.432	0.13	0.66	0.20	0.842
**Other companion animal guardian** (0=No)												
Yes	-0.56	0.79	-0.71	0.478	0.05	0.14	0.33	0.742	0.51	0.64	0.79	0.427

Table S2 (cont.)

	Anxiety (n = 1049)				Stress (n = 1034)			
	$\hat{\beta}$	SE	t	*p*	$\hat{\beta}$	SE	t	*p*
**Step 1**	$R^2 = 0.071$ $\chi^2 (12) = 83.47$, $p < 0.001$				$R^2 = 0.084$ $\chi^2 (12) = 91.39$, $p < 0.001$			
Intercept	6.40	0.50	12.81	<0.001	14.35	0.91	15.73	<0.001
**Age** (0=18-34 years)								
35-50 years	**-1.37**	**0.36**	**-3.83**	**<0.001**	**-3.30**	**0.66**	**-5.03**	**<0.001**
51-69 years	**-2.02**	**0.38**	**-5.26**	**<0.001**	**-4.69**	**0.71**	**-6.59**	**<0.001**
70+ years	**-2.48**	**0.76**	**-3.28**	**0.001**	**-6.57**	**1.40**	**-4.70**	**<0.001**
**Gender** (0=Female)								
Male	**-1.19**	**0.38**	**-3.12**	**0.002**	**-1.83**	**0.70**	**-2.59**	**0.010**
**Highest level of education** (0=School or below)								
Undergraduate	-0.72	0.47	-1.54	0.123	-0.60	0.85	-0.70	0.484
Masters	**-1.22**	**0.49**	**-2.51**	**0.012**	0.17	0.89	0.19	0.850
Doctorate	**-1.31**	**0.53**	**-2.49**	**0.013**	-0.51	0.97	-0.53	0.597
**Marital status** (0=Single, separated, divorced, widowed)								
Partnered, not living together	1.00	0.64	1.58	0.116	1.39	1.14	1.21	0.226
Married or living with partner	**-0.72**	**0.32**	**-2.27**	**0.023**	0.13	0.59	0.22	0.823
**Live with children** (0=No)								
Yes	-0.13	0.36	-0.36	0.720	0.44	0.67	0.66	0.511
**Country** (0=United Kingdom)								
United States	0.07	0.34	0.20	0.840	-0.81	0.62	-1.30	0.192
Other	-0.66	0.41	-1.61	0.108	**-1.90**	**0.76**	**-2.52**	**0.012**

	Anxiety (n = 1049)				Stress (n = 1034)			
	$\hat{\beta}$	SE	t	*p*	$\hat{\beta}$	SE	t	*p*
**Step 2**	$\Delta R^2 = 0.026$ $\chi^2 (8) = 31.37$, $p < 0.001$				$\Delta R^2 = 0.026$ $\chi^2 (8) = 27.66$, $p < 0.001$			
Intercept	6.40	0.69	9.21	<0.001	15.60	1.25	12.50	<0.001
**Age** (0=18-34 years)								
35-50 years	**-1.20**	**0.36**	**-3.34**	**0.001**	-3.08	**0.65**	**-4.72**	**<0.001**

51-69 years	**-1.99**	**0.39**	**-5.08**	**<0.001**	-4.53	**0.71**	**-6.35**	**<0.001**
70+ years	**-2.93**	**0.81**	**-3.62**	**<0.001**	-6.55	**1.46**	**-4.48**	**<0.001**
**Gender** (0=Female)								
Male	**-1.17**	**0.38**	**-3.06**	**0.002**	-1.80	**0.70**	**-2.59**	**0.010**
**Highest level of education** (0=School or below)								
Undergraduate	-0.59	0.47	-1.27	0.206	-0.38	0.84	-0.45	0.652
Masters	**-1.07**	**0.49**	**-2.17**	**0.030**	0.18	0.90	0.20	0.845
Doctorate	**-1.06**	**0.54**	**-1.97**	**0.049**	-0.25	0.98	-0.26	0.797
**Marital status** (0=Single, separated, divorced, widowed)								
Partnered, not living together	1.10	0.64	1.73	0.085	1.31	1.13	1.16	0.246
Married or living with partner	-0.61	0.32	-1.89	0.060	0.07	0.58	0.12	0.907
**Live with children** (0=No)								
Yes	-0.13	0.36	-0.36	0.719	0.46	0.66	0.69	0.488
**Country** (0=United Kingdom)								
United States	-0.28	0.35	-0.80	0.425	**-1.29**	**0.64**	**-2.03**	**0.043**
Other	-0.77	0.42	-1.85	0.065	**-2.21**	**0.76**	**-2.91**	**0.004**
**Communicated via verbal means** (0=Average)								
Less than average	0.59	0.35	1.67	0.095	1.16	0.64	1.81	0.070
More than average	**0.81**	**0.34**	**2.37**	**0.018**	**1.28**	**0.62**	**2.05**	**0.041**
**Communicated face-to-face** (0=Average)								
Less than average	0.21	0.38	0.57	0.572	-0.32	0.69	-0.47	0.639
More than average	0.06	0.37	0.17	0.866	**-1.60**	**0.66**	**-2.41**	**0.016**
**Left the house to exercise** (0=Average)								
Less than average	-0.16	0.40	-0.41	0.681	-0.99	0.72	-1.38	0.169
More than average	**-0.89**	**0.36**	**-2.50**	**0.013**	**-2.10**	**0.65**	**-3.25**	**0.001**
**Working during pandemic** (0=No)								
Yes	-0.59	0.38	-1.57	0.116	-0.28	0.68	-0.41	0.684
**Period of isolation >1 week** (0=No)								
Yes	**1.12**	**0.33**	**3.42**	**0.001**	0.95	0.59	1.61	0.107
	Anxiety (n = 1049)				Stress (n = 1034)			
	$\hat{\beta}$	SE	t	*p*	$\hat{\beta}$	SE	t	*p*
**Step 3**	$\Delta R^2 = 0.010$ $\chi^2(5) = 9.88$, $p = 0.079$				$\Delta R^2 = 0.003$ $\chi^2(5) = 3.27$, $p = 0.658$			
Intercept	6.02	0.78	7.68	<0.001	14.57	1.42	10.29	<0.001

**Age** (0=18-34 years)								
35-50 years	**-1.16**	**0.36**	**-3.21**	**0.001**	**-3.11**	**0.66**	**-4.74**	**<0.001**
51-69 years	**-1.91**	**0.39**	**-4.86**	**<0.001**	**-4.51**	**0.72**	**-6.27**	**<0.001**
70+ years	**-2.74**	**0.81**	**-3.36**	**0.001**	**-6.32**	**1.48**	**-4.28**	**<0.001**
**Gender** (0=Female)								
Male	**-1.20**	**0.39**	**-3.08**	**0.002**	**-1.67**	**0.71**	**-2.34**	**0.019**
**Highest level of education** (0=School or below)								
Undergraduate	-0.52	0.47	-1.11	0.265	-0.32	0.85	-0.37	0.710
Masters	-0.93	0.50	-1.87	0.062	0.36	0.91	0.40	0.690
Doctorate	-0.91	0.54	-1.68	0.094	-0.07	0.99	-0.07	0.942
**Marital status** (0=Single, separated, divorced, widowed)								
Partnered, not living together	1.02	0.64	1.59	0.113	1.35	1.14	1.18	0.238
Married or living with partner	-0.63	0.32	-1.94	0.052	<0.01	0.59	<0.01	0.999
**Live with children** (0=No)								
Yes	-0.31	0.37	-0.83	0.406	0.38	0.67	0.56	0.574
**Country** (0=United Kingdom)								
United States	-0.25	0.36	-0.68	0.494	**-1.42**	**0.66**	**-2.16**	**0.031**
Other	-0.68	0.43	-1.59	0.113	**-2.28**	**0.79**	**-2.90**	**0.004**
**Communicated via verbal means** (0=Average)								
Less than average	0.57	0.35	1.63	0.104	1.10	0.64	1.71	0.088
More than average	**0.92**	**0.34**	**2.68**	**0.008**	**1.39**	**0.63**	**2.21**	**0.028**
**Communicated face-to-face** (0=Average)								
Less than average	0.20	0.38	0.53	0.594	-0.28	0.69	-0.41	0.682
More than average	0.01	0.37	0.03	0.980	**-1.62**	**0.67**	**-2.42**	**0.016**
**Left the house to exercise** (0=Average)								
Less than average	-0.22	0.40	-0.55	0.581	-1.04	0.72	-1.44	0.150
More than average	**-0.82**	**0.37**	**-2.25**	**0.025**	**-2.14**	**0.67**	**-3.20**	**0.001**
**Working during pandemic** (0=No)								
Yes	-0.54	0.38	-1.42	0.156	-0.25	0.69	-0.37	0.713
**Period of isolation >1 week** (0=No)								
Yes	**1.09**	**0.33**	**3.30**	**0.001**	0.90	0.59	1.51	0.131
**Companion animal guardian** (0=No)								
Yes	-0.20	0.57	-0.36	0.720	0.88	1.05	0.84	0.400
**Dog guardian** (0=No)								
Yes	-0.01	0.38	-0.03	0.980	<0.01	0.70	<0.01	0.996
**Cat guardian** (0=No)								

Yes	0.33	0.35	0.94	0.349	0.29	0.64	0.45	0.656
**Fish guardian** (0=No)								
Yes	0.63	0.39	1.60	0.111	<0.01	0.72	<0.01	1.000
**Other companion animal guardian** (0=No)								
Yes	**0.81**	**0.38**	**2.15**	**0.032**	0.65	0.68	0.94	0.345

Note: $\hat{\beta}$ are unstandardised regression coefficients, results in bold are statistically significant at $p < 0.05$.

Table S3. Results of robust hierarchical regression analyses for level of engagement with companion animals.

	Mental well-being				UCLA Loneliness Scale				Depression			
	$\hat{\beta}$	SE	t	p	$\hat{\beta}$	SE	t	p	$\hat{\beta}$	SE	t	p
**Fishes**	$R^2 = 0.029$<br>$\chi^2(10) = 5.37$, $p = 0.865$, n = 200				$R^2 = 0.048$<br>$\chi^2(10) = 9.45$, $p = 0.450$, n = 208				$R^2 = 0.026$<br>$\chi^2(10) = 5.33$, $p = 0.868$, n=204			
Intercept	44.67	4.74	9.42	<0.001	5.59	0.88	6.35	<0.001	8.58	3.99	2.15	0.033
**Primary caregiver** (0=No)												
Responsibility equally shared with another	2.22	4.05	0.55	0.585	-0.20	0.75	-0.27	0.788	-0.60	3.40	-0.18	0.860
Yes	-0.34	3.91	-0.09	0.931	0.07	0.73	0.09	0.926	1.03	3.31	0.31	0.756
**Time spent daily on tank/pond maintenance** (0= An average amount)												
Less than average	0.47	2.29	0.20	0.838	-0.17	0.42	-0.40	0.690	2.48	1.91	1.30	0.196
More than average	0.90	2.19	0.41	0.683	-0.44	0.41	-1.08	0.282	-0.68	1.83	-0.37	0.712
**Time spent daily feeding fishes** (0= An average amount)												
Less than average	2.07	4.14	0.50	0.618	-0.61	0.77	-0.79	0.431	-1.21	3.47	-0.35	0.727
More than average	0.66	1.99	0.33	0.740	0.10	0.37	0.27	0.791	-0.42	1.68	-0.25	0.801
**Time spent daily watching fishes** (0= An average amount)												
Less than average	2.22	2.28	0.97	0.332	0.08	0.43	0.19	0.848	-2.51	1.92	-1.31	0.193
More than average	-1.04	2.10	-0.49	0.623	0.18	0.39	0.46	0.647	0.01	1.75	0.01	0.996
**Time spent daily talking to fishe**s (0= An average amount)												
Less than average	0.26	2.06	0.13	0.900	**-0.76**	**0.38**	**-1.98**	**0.049**	0.87	1.74	0.50	0.618
More than average	0.90	2.28	0.39	0.694	-0.46	0.42	-1.08	0.280	0.68	1.91	0.36	0.721

	Mental well-being				UCLA Loneliness Scale				Depression			
	$\hat{\beta}$	SE	t	p	$\hat{\beta}$	SE	t	p	$\hat{\beta}$	SE	t	p
**Dogs**	$R^2 = 0.053$<br>$\chi^2(12) = 30.65$, $p = 0.002$, n = 581				$R^2 = 0.063$<br>$\chi^2(12) = 38.41$, $p < 0.001$, n = 609				$R^2 = 0.056$<br>$\chi^2(12) = 34.89$, $p < 0.001$, n = 592			

Intercept	39.48	2.62	15.08	<0.001	5.40	0.46	11.71	<0.001	14.17	2.12	6.67	<0.001
**Primary caregiver** (0=No)												
Responsibility equally shared with another	**7.82**	**2.27**	**3.45**	**0.001**	**-1.33**	**0.40**	**-3.35**	**0.001**	**-6.46**	**1.84**	**-3.50**	**0.001**
Yes	**6.97**	**2.28**	**3.06**	**0.002**	**-0.92**	**0.40**	**-2.31**	**0.021**	**-6.73**	**1.85**	**-3.64**	**<0.001**
**Time spent daily walking dogs** (0= An average amount)												
Less than average	-0.92	0.97	-0.95	0.343	**0.41**	**0.17**	**2.35**	**0.019**	1.05	0.80	1.31	0.189
More than average	-0.20	1.05	-0.19	0.851	0.22	0.19	1.16	0.245	0.25	0.86	0.29	0.775
**Time spent daily feeding dogs** (0= An average amount)												
Less than average	1.75	1.00	1.76	0.080	-0.06	0.18	-0.36	0.716	-0.33	0.81	-0.41	0.685
More than average	0.34	1.06	0.32	0.748	-0.08	0.19	-0.45	0.651	-0.72	0.87	-0.84	0.403
**Time spent daily petting dogs** (0= An average amount)												
Less than average	-0.33	1.25	-0.27	0.790	-0.08	0.22	-0.36	0.723	-0.91	1.02	-0.89	0.374
More than average	**-2.22**	**1.08**	**-2.07**	**0.039**	0.09	0.19	0.45	0.656	**1.78**	**0.88**	**2.03**	**0.043**
**Time spent daily talking to dogs** (0= An average amount)												
Less than average	**2.41**	**1.22**	**1.97**	**0.050**	-0.33	0.22	-1.49	0.138	-1.28	1.00	-1.28	0.202
More than average	1.02	1.19	0.85	0.395	0.14	0.21	0.66	0.513	-0.08	0.98	-0.08	0.936
**Time spent daily on other activities with dogs** (0=An average amount)												
Less than average	-1.80	1.15	-1.56	0.119	**0.49**	**0.20**	**2.40**	**0.017**	0.97	0.94	1.03	0.302
More than average	-0.31	1.10	-0.28	0.778	0.34	0.20	1.71	0.087	0.74	0.90	0.82	0.413

	Mental well-being				UCLA Loneliness Scale				Depression			
	$\hat{\beta}$	SE	t	*p*	$\hat{\beta}$	SE	t	*p*	$\hat{\beta}$	SE	t	*p*
**Cats**	$R^2 = 0.036$ $\chi^2(10) = 14.24$, $p = 0.162$, n = 415				$R^2 = 0.035$ $\chi^2(10) = 14.83$, $p = 0.139$, n = 442				$R^2 = 0.052$ $\chi^2(10) = 23.39$, $p = 0.009$, n = 429			
Intercept	43.88	2.77	15.85	<0.001	5.57	0.54	10.31	<0.001	12.65	2.28	5.54	<0.001
**Primary caregiver** (0=No)												
Responsibility equally shared with another	2.04	2.26	0.90	0.369	-0.76	0.44	-1.72	0.086	-2.78	1.86	-1.49	0.137
Yes	0.74	2.25	0.33	0.742	-0.28	0.44	-0.62	0.535	-2.04	1.86	-1.10	0.273
**Time spent daily feeding cats** (0= An average amount)												
Less than average	1.76	1.16	1.52	0.130	-0.17	0.22	-0.78	0.437	-0.29	0.95	-0.31	0.761
More than average	-0.42	1.34	-0.32	0.751	-0.06	0.25	-0.25	0.803	0.13	1.11	0.12	0.907
**Time spent daily petting cats**(0= An average amount)												
Less than average	-0.46	1.31	-0.35	0.724	0.13	0.25	0.52	0.603	1.42	1.08	1.32	0.188
More than average	-1.05	1.42	-0.74	0.461	0.46	0.27	1.69	0.093	1.31	1.18	1.11	0.268
**Time spent daily talking to cats** (0= An average amount)												
Less than average	2.58	1.40	1.85	0.065	-0.38	0.27	-1.43	0.154	**-4.29**	**1.15**	**-3.73**	**<0.001**
More than average	1.14	1.48	0.77	0.441	-0.49	0.28	-1.72	0.087	-2.42	1.23	-1.96	0.050
**Time spent daily on other activities with cats** (0= An average amount)												

Less than average	-1.50	1.38	-1.09	0.278	0.15	0.26	0.59	0.558	0.24	1.13	0.21	0.833
More than average	-0.43	1.50	-0.29	0.772	0.07	0.28	0.26	0.796	0.66	1.22	0.54	0.591

Table S3 (cont.)

	Anxiety				Stress			
	$\hat{\beta}$	SE	t	*p*	$\hat{\beta}$	SE	t	*p*
**Fishes**	$R^2 = 0.036$; χ^2 (10) = 7.34, *p* = 0.693, n = 203				$R^2 = 0.047$; χ^2 (10) = 9.46, *p* = 0.489, n = 201			
Intercept	4.81	2.52	1.91	0.058	18.87	3.90	4.84	<0.001
**Primary caregiver** (0=No)								
Responsibility equally shared with another	-0.29	2.16	-0.13	0.895	-5.11	3.32	-1.54	0.126
Yes	0.96	2.11	0.45	0.650	-3.33	3.20	-1.04	0.299
**Time spent daily on tank/pond maintenance** (0= An average amount)								
Less than average	-0.67	1.20	-0.55	0.581	-2.46	1.87	-1.31	0.191
More than average	-0.99	1.16	-0.86	0.391	-2.78	1.80	-1.54	0.125
**Time spent daily feeding fishes** (0= An average amount)								
Less than average	-0.97	2.21	-0.44	0.661	-1.21	3.37	-0.36	0.721
More than average	0.19	1.05	0.18	0.856	-0.70	1.64	-0.43	0.671
**Time spent daily watching fishes** (0= An average amount)								
Less than average	0.37	1.22	0.30	0.766	-1.67	1.86	-0.90	0.369
More than average	0.88	1.11	0.80	0.427	-0.78	1.73	-0.45	0.651
**Time spent daily talking to fishes** (0= An average amount)								
Less than average	-1.21	1.09	-1.11	0.268	-2.26	1.69	-1.34	0.183
More than average	-0.49	1.19	-0.41	0.679	-0.09	1.87	-0.05	0.961

	Anxiety				Stress			
	$\hat{\beta}$	SE	t	*p*	$\hat{\beta}$	SE	t	*p*
**Dogs**	$R^2 = 0.071$; χ^2 (12) = 48.03, *p* <0.001, n = 592				$R^2 = 0.048$; χ^2 (12) = 28.19, *p* = 0.005, n = 587			
Intercept	6.36	1.20	5.31	<0.001	16.25	2.23	7.30	<0.001
**Primary caregiver** (0=No)								
Responsibility equally shared with another	**-2.80**	**1.03**	**-2.70**	**0.007**	**-4.38**	**1.91**	**-2.29**	**0.022**

Yes	**-3.03**	**1.04**	**-2.93**	**0.004**	**-6.04**	**1.92**	**-3.15**	**0.002**
**Time spent daily walking dogs** (0= An average amount)								
Less than average	**1.38**	**0.46**	**3.02**	**0.003**	1.06	0.85	1.24	0.215
More than average	-0.06	0.49	-0.13	0.898	-0.46	0.92	-0.51	0.614
**Time spent daily feeding dogs** (0= An average amount)								
Less than average	0.34	0.46	0.74	0.461	-0.55	0.87	-0.63	0.529
More than average	0.16	0.50	0.32	0.749	-0.25	0.93	-0.27	0.784
**Time spent daily petting dogs** (0= An average amount)								
Less than average	**-1.59**	**0.58**	**-2.74**	**0.006**	-1.33	1.10	-1.21	0.226
More than average	-0.19	0.51	-0.37	0.711	0.62	0.94	0.66	0.511
**Time spent daily talking to dogs** (0= An average amount)								
Less than average	-0.89	0.57	-1.56	0.120	-1.57	1.07	-1.46	0.144
More than average	-0.51	0.56	-0.91	0.363	-1.30	1.04	-1.25	0.213
**Time spent daily on other activities with dogs** (0=An average amount)								
Less than average	0.17	0.54	0.31	0.754	0.38	1.01	0.38	0.704
More than average	0.95	0.52	1.83	0.067	1.35	0.97	1.40	0.162

	Anxiety				Stress			
	$\hat{\beta}$	SE	t	*p*	$\hat{\beta}$	SE	t	*p*
**Cats**	$R^2 = 0.045$ $\chi^2(10) = 20.48$, $p = 0.025$, n = 430				$R^2 = 0.049$ $\chi^2(10) = 21.25$, $p = 0.019$, n = 428			
Intercept	4.94	1.41	3.51	<0.001	15.76	2.53	6.23	<0.001
**Primary caregiver** (0=No)								
Responsibility equally shared with another	-1.12	1.16	-0.96	0.336	-2.73	2.07	-1.32	0.188
Yes	-0.83	1.16	-0.72	0.474	-3.65	2.07	-1.77	0.078
**Time spent daily feeding cats** (0= An average amount)								
Less than average	0.40	0.58	0.70	0.486	-1.05	1.04	-1.02	0.310
More than average	-0.08	0.68	-0.12	0.904	0.04	1.21	0.04	0.972
**Time spent daily petting cats**(0= An average amount)								
Less than average	1.19	0.65	1.83	0.068	1.32	1.17	1.13	0.261
More than average	1.34	0.71	1.88	0.061	1.45	1.29	1.13	0.260
**Time spent daily talking to cats** (0= An average amount)								
Less than average	-1.35	0.70	-1.94	0.053	**-3.65**	**1.25**	**-2.92**	**0.004**
More than average	**-1.76**	**0.75**	**-2.35**	**0.019**	**-3.01**	**1.35**	**-2.23**	**0.026**

**Time spent daily on other activities with cats** (0= An average amount)								
Less than average	-0.73	0.68	-1.07	0.285	0.07	1.23	0.06	0.956
More than average	0.96	0.74	1.29	0.197	1.39	1.34	1.04	0.299

Note: $\hat{\beta}$ are unstandardised regression coefficients, results in bold are statistically significant at $p < 0.05$
